# Enhancing supercapacitor performance of Ni–Co–Mn metal–organic frameworks by compositing it with polyaniline and reduced graphene oxide

**DOI:** 10.1039/d3ra07788h

**Published:** 2024-01-09

**Authors:** Mohsin Ali Marwat, Shaheer Ishfaq, Kanwar Muhammad Adam, Bilal Tahir, Muhammad Hamza Shaikh, Muhammad Fawad Khan, Muhammad Ramzan Abdul Karim, Zia Ud Din, Syed Abdullah, Esha Ghazanfar

**Affiliations:** a Faculty of Materials and Chemical Engineering, Ghulam Ishaq Khan (GIK) Institute of Engineering Sciences and Technology Topi 23640 Pakistan mohsin.ali@giki.edu.pk +92-938-281032 +92-938-281026

## Abstract

Metal–organic frameworks (MOFs) are one of the most sought-after materials in the domain of supercapacitors and can be tailored to accommodate diverse compositions, making them amenable to facile functionalization. However, their intrinsic specific capacitance as well as energy density is minimal, which hinders their usage for advanced energy storage applications. Therefore, herein, we have prepared six electrodes, *i.e.*, Ni–Co–Mn MOFs, polyaniline (PANI), and reduced graphene oxide (rGO) along with their novel nanocomposites, *i.e.*, C_1_, C_2_, and C_3_, comprising MOFs : PANI : rGO in a mass ratio of 100 : 1 : 0.5, 100 : 1 : 1, and 100 : 1 : 10, respectively. The polyaniline conducting polymer and rGO enabled efficient electron transport, enhanced charge storage processes, substantial surface area facilitating higher loading of active materials, promoting electrochemical reactions, and ultimately enhanced nanocomposite system performance. As a result, scanning electron microscopy (SEM) and X-ray diffraction (XRD) techniques confirmed the successful synthesis and revealed distinct morphological features of the materials. Following electrochemical testing, it was observed that composition C_2_ exhibited the highest performance, demonstrating a groundbreaking specific capacitance of 1007 F g^−1^ at 1 A g^−1^. The device showed a good energy density of 25.11 W h kg^−1^ and a power density of 860 W kg^−1^. Remarkably, the device demonstrated a capacity retention of 115% after 1500 cycles, which is a clear indication of the wettability factor, according to the literature. The power law indicated *b*-values in a range of 0.58–0.64, verifying the hybrid-type behavior of supercapacitors.

## Abstract 2

NiCoMn metal–organic frameworks (MOFs) are one of the most sought-after materials in the domain of supercapacitors. Here, they are composited with rGO and polyaniline to increase surface area and conductivity, aiming for enhanced energy density.

## Graphical abstract

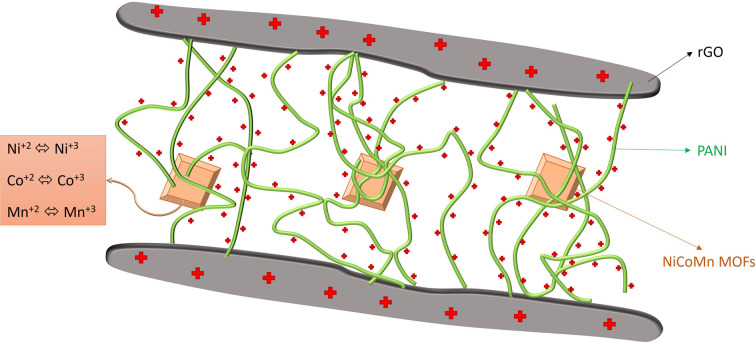

## 1. Introduction

The unstoppable progression of technological development has led us into an era marked by an insatiable demand for energy.^1–5^ With our growing dependence on complex technological systems, there is an escalating need to improve the capacity and efficiency of energy storage.^6–8^ Consequently, researchers are actively investigating innovative methods to create advanced energy storage devices that can store larger amounts of energy that can be used over extended periods. Significantly, considerable resources are currently being allocated to the exploration of cutting-edge materials for energy storage purposes.^9–12^ This includes a focus on flexible capacitors, supercapacitors, batteries, and energy conversion devices like fuel cells, solar cells, thermoelectric units, and wind turbines.^13–16^

Supercapacitors have emerged as a prominent and promising technology, acquiring considerable attention due to their exceptional characteristics.^17,18^ Their impressive attributes, such as high power density, extended cycle life, rapid charging capabilities, and remarkable efficiency, surpass those of other available energy storage devices.^19^ These features coupled with the ability to combine supercapacitors with other energy storage devices, such as batteries, to create hybrid supercapattery systems further establish supercapacitors as a superior alternative in the field of energy storage.^20^ The primary obstacle impeding the commercialization of supercapacitors is their low energy density.^21^ EDLC, *i.e.*, an electric double layer capacitor, refers to a supercapacitor type that exploits the ion absorption phenomenon occurring at the double-layer interface for the purpose of energy storage in supercapacitors of the EDLC type.^22^ Despite their advantages such as high power density, excellent cyclability, high efficiency, and broad operating temperature range, these devices encounter challenges associated with low energy density, self-discharge, and voltage limitations. Conversely, pseudocapacitive supercapacitors, which rely on faradaic reactions for energy storage, exhibit drawbacks including reduced cyclic life, and lower power densities, alongside notable advantages such as high specific capacitance and superior energy densities.^23–25^

The synergistic combination of these two phenomena presents an opportunity to create a hybrid device that can effectively enhance energy densities while simultaneously preserving power density, rate capability, and cyclability. To accomplish this, a dual-structured architecture is employed, wherein one component of the hybrid device operates *via* EDLC behaviour, leveraging its inherent strengths, while the other component functions as a pseudocapacitor, harnessing its distinctive properties.^26^ This integration of EDLC and pseudocapacitor functionalities within the hybrid device enables the realization of improved energy storage performance without compromising other critical parameters.^27,28^ The development of nanocomposites emerges as a captivating trajectory for augmenting the synergistic amalgamation of electric double layer capacitance (EDLC) and pseudocapacitive behaviour within hybrid supercapacitors. By virtue of their custom-engineered composition and distinct structural attributes, nanocomposites present remarkable prospects for optimizing electrochemical performance, namely the attainment of significantly augmented surface area, enhanced conductivity, and a greater abundance of active sites conducive to charge storage. It is noteworthy that this domain has garnered substantial attention from researchers, warranting deeper contemplation and meticulous investigation.^29^

Metal–organic frameworks (MOFs) represent a burgeoning category of chemical compounds exhibiting a distinctive structural arrangement, characterized by the interconnectedness of inorganic nodes *via* organic linkers.^30,31^ These crystalline entities have garnered substantial attention owing to their remarkable porous architecture, which imparts them with exceptional qualities. Most notably, MOFs possess high porosity and possess an extensive surface area, attributes that contribute to their versatility. MOFs can be tailored to accommodate a diverse range of compositions, rendering them amenable to facile functionalization. Consequently, these materials have found wide-ranging applications in numerous scientific domains, including catalysis, gas separation and storage, drug delivery, ion sensing, optoelectronics, and charge storage.^32^ MOFs, *i.e.*, metal–organic frameworks and their derived nanostructures received significant attention for their extraordinary energy storage performance in the field of supercapacitors. The exceptional porosity exhibited by MOFs grants them a substantial capacity for hosting charge, rendering them highly attractive for energy storage applications. Nevertheless, the presence of organic linkers poses a crucial limitation by impeding efficient charge transport and hindering conductivity.^33^ A viable approach to overcoming this limitation would be to combine MOFs with other conductive materials in a nanocomposite.

The utilization of conducting polymers (CPs) as materials for fabricating nanocomposites holds significant promise. This is primarily attributed to their possession of several desirable characteristics akin to conventional polymers, including cost-effectiveness, facile synthesis, exceptional mechanical flexibility, lightweight nature, and commendable environmental stability, while also possessing good intrinsic conductivity akin to metals and semiconductors.^34^ Polyaniline (PANI) has garnered significant attention within the realm of conducting polymers (CPs) as an electrode owing to exhibiting distinctive redox states, excellent thermal stability, low cost, and remarkable theoretical capacitance.^35^ Its cyclability as an electrode in a supercapacitor, however, suffers due to swelling/shrinkage changes,^36^ which results in mechanical degradation caused by the doping/undoping of anions or cations during operation. Another problem is the diminishing conductivity of the polymer chains further exacerbating the decline in its specific capacitance over time. An additional constraint arises from the divergence observed between the experimental specific capacitance of polyaniline (PANI) and its theoretical value, attributable to localized redox reactions predominantly confined to the surface of the polymer within a few nanometers, rather than propagating into its bulk. PANI is also susceptible to material loss because of its tendency to peel off from the active material, thereby exacerbating the cyclability issues encountered by the electrode.^37^ To address these challenges, a promising approach involves the integration of polyaniline (PANI) with carbon-based materials. Carbon materials exhibit notable attributes such as exceptional chemical stability, superior mechanical durability, cost-effectiveness in synthesis, and wide-ranging conductivity at various temperatures. By combining PANI with carbon-based materials, a stable network can be formed to provide structural support for PANI, while simultaneously facilitating efficient charge transport through well-established conductive pathways. This composite strategy enables enhanced stability, improved cyclability, and increased charge transport rates, thus offering a viable solution to mitigate the limitations associated with PANI as an electrode material.^38^

In the realm of carbon-based materials, such as carbon nanotubes (CNTs), carbon nano-onions (CNOs), carbon nanofibers (CNFs), graphene, graphene oxide, and reduced graphene oxide (rGO), which have demonstrated promising performance in supercapacitors,^39–42^ reduced graphene oxide (rGO) distinguishes itself among carbon-based materials due to its distinctive structural characteristics, namely a two-dimensional sp^2^ carbon lattice. This unique arrangement confers rGO with exceptional electrical conductivity and facilitates efficient electron transport, thereby enhancing charge storage and transfer processes. The sp^2^ carbon network is restored during the reduction of GO, resulting in the removal of oxygen-containing functional groups, which in turn boosts the material's conductivity. rGO also possesses a substantial specific surface area, which enables higher loading of active materials and facilitates electrochemical reactions.^43^ The presence of defects and functional groups on the surface of rGO enhances its interaction with PANI or other active materials, leading to an overall improvement in the performance of composite systems.^44^

In this study, we present the development of a novel nanocomposite composition aimed at achieving enhanced charge storage capabilities for supercapacitor applications. The composition involved the combination of tertiary Ni–Co–Mn metal–organic frameworks (MOFs) with polyaniline (PANI) and reduced graphene oxide (rGO). Initially, we individually evaluated the electrochemical performance of the base materials, namely pristine Ni–Co–Mn MOFs (MOFs), PANI, and rGO, by fabricating electrodes on Ni-foam substrates. Subsequently, three compositions were prepared by varying the ratios of the base materials. Composition C_1_ consisted of MOFs : rGO : PANI at a mass ratio of 100 : 1 : 0.5, C_2_ at 100 : 1 : 1, and C_3_ at 100 : 1 : 10. Following electrochemical testing, it was observed that composition C_2_ exhibited the highest performance, demonstrating a specific capacitance of 1007 F g^−1^ at 1 A g^−1^ in galvanostatic charge–discharge (GCD) measurements. Additionally, C_2_ displayed the longest discharge time in GCD and exhibited the highest redox peaks in cyclic voltammetry (CV). To further evaluate its practical application, an asymmetric supercapacitor device was fabricated utilizing the C_2_ electrode in an electrolyte of 1 M KOH. This device exhibited 70.625 F g^−1^ of a specific capacitance at 1 A g^−1^ of a current density within 0–1.6 V of a potential window, leading to 25.11 W h kg^−1^ and 860 W h kg^−1^ of energy and power densities. Remarkably, the retention of the specific capacity of the device reached 115% after 1500 cycles. This can be attributed to the improved wettability of the electrode surface and the reopening of previously clogged pores due to repeated cycling, thereby increasing the affinity for the reaction. Also, the observed *b* values corroborate our assertion of a hybrid supercapacitor.

## 2. Experimental

### 2.1. Materials

Laboratory-grade pure 1-methyl-2-pyrrolidone (NMP) as a solvent and hexamethylenetetramine (HMT) as an organic ligand were acquired from Honeywell (Germany) and used as received without further purification or modification. Flake graphite (High carbon), sodium nitrate (NaNO_3_), cobalt(ii) nitrate hexahydrate (Co(NO_3_)_2_·6H_2_O), manganese(ii) nitrate tetrahydrate (Mg(NO_3_)_2_·6H_2_O), nickel(ii) nitrate hexahydrate (Ni(NO_3_)_2_·6H_2_O), potassium permanganate (KMnO_4_), hydrogen peroxide (H_2_O_2_), aniline (C_6_H_5_NH_2_), 37% hydrochloric acid (HCl) and 98% sulphuric acid (H_2_SO_4_), were used as-received, and were acquired from a well-known company, *i.e.*, Sigma Aldrich. DI water, acetone, and ethanol were acquired locally from Sigma Scientific Private Ltd. and were used for cleaning purposes.

### 2.2. Synthesis of MOFs

**Fig. 1:**
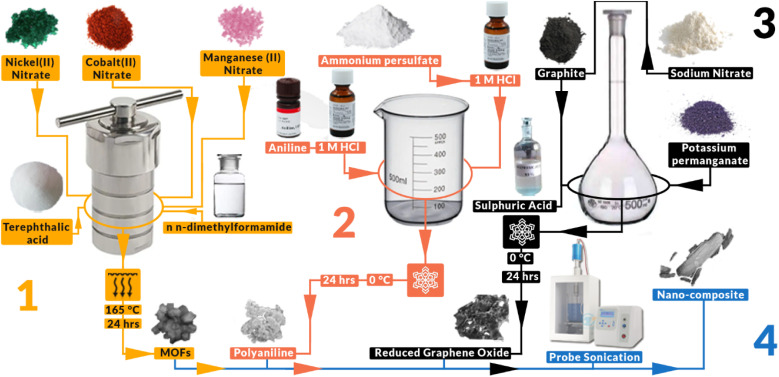
An illustrative graph outlining the experimental procedure undertaken in this project. The figure visually depicts the step-by-step process for synthesizing each individual constituent, followed by the methodology employed to fabricate the nanocomposite material.

7 mmol of Co(ii) nitrate hexahydrate (0.29103 g), 7 mmol of Mn(ii) nitrate tetrahydrate (0.25101 g) and 7 mmol of Ni(ii) nitrate hexahydrate (0.29081 g) were added into a beaker along with 5 mmol of terephthalic acid as ligand and 60 mL of *N*,*N*-dimethylformamide (DMF) as the solvent. After stirring for 30 min on a magnetic stirred this solution was then sealed into a Teflon lined autoclave and placed into an oven at 165 °C for 24 hours, as shown in Fig. 1. The resulting solution and particles were then taken out and centrifuged for 10 min at 3000 rpm. This was followed by a washing cycle with DI water followed by ethanol until the particles were clean. The resulting MOFs were then dried in air at 80 °C for 24 hours.

### 2.3. Preparation of polyaniline (PANI)

The synthesis of polyaniline (PANI) was performed by stirring 1 g of pure aniline (ANI) with 12.5 mL of 1 M hydrochloric acid (HCl) solution in an ice bath, followed by the slow addition of 30 L acid-saturated solution of ammonium persulfate (APS) with a constant 1 : 1 molar ratio of ANI to APS, as shown in Fig. 1. The precipitated product was collected after magnetically stirring a reaction mixture for 6 hours. Then it was washed with distilled water until neutralized and dried overnight. The product was purified with acetone and dried at 80 °C for 24 hours.

### 2.4. Preparation of reduced graphene oxide (rGO)

Graphite flakes (2 g) were subjected to bath sonication at a frequency of 20 kHz, followed by their combination with 2 g of NaNO_3_ in 90 mL of H_2_SO_4_ (98%) within a 1000 mL volumetric flask. The mixture was continuously stirred in an ice bath, maintaining a temperature range of 0–5 °C, for a duration of four hours, as shown in Fig. 1. Throughout this process, utmost care was taken to ensure the gradual addition of potassium permanganate (12 g) to the suspension, with precise control over the addition rate to maintain the reaction temperature below 15 °C. Following thorough stirring for 30 minutes to ensure proper dispersion, the mixture was heated to 35 °C using a heating mantle. A discernible brownish taint was observed in the solution, which was stirred for a duration of 24 hours at this temperature. Subsequently, 100 mL of water was added very slowly, resulting in an increase in temperature due to the exothermic nature of the water–sulfuric acid reaction. The heating mantle was then adjusted to raise the temperature to 98 °C, leading to the solution turning brown and the occurrence of effervescence. After the cessation of bubbles, 200 mL of deionized (DI) water was added to further dilute the acid. To terminate the reaction, 10 mL of H_2_O_2_ was added, resulting in the appearance of yellow colour in the solution. The solution was then subjected to filtration and washed with DI water until a neutral pH was attained. The obtained product was a brown powder identified as graphene oxide (GO). The prepared powder was then annealed at 300 °C for 6 hours at a heating rate of 2 °C per min to obtain rGO powder.

### 2.5. Fabrication of nanocomposites

In two separate beakers, a specific mass of metal–organic frameworks (MOFs) and polyaniline (PANI) were individually combined with 10 mL of deionized (DI) water and stirred thoroughly for 30 minutes. Simultaneously, reduced graphene oxide (rGO) was subjected to bath sonication at a frequency of 20 kHz for 30 minutes. Subsequently, the MOF solution was positioned beneath the probe of an Ultra Probe Sonicator, which was activated at 30% power, and the PANI solution was gradually added over a period of 5 minutes. After the mixing of both solutions, probe sonication was carried out for 10 minutes. Following this, the rGO was slowly introduced over a duration of 5 minutes, and the solution was subjected to an additional 10 minutes of probe sonication to obtain the nanocomposite-containing solution, as shown in Fig. 1. The various mass ratios of the materials were designated as C_1_ (MOFs : PANI : rGO at a mass ratio of 100 : 1 : 0.5), C_2_ (at 100 : 1 : 1), and C_3_ (at 100 : 1 : 10); see Table 1.

**Table 1:** List of the compositions of composites

S. no.	Sample	MOFs	PANI	rGO
1	C_1_	100	1	0.5
2	C_1_	100	1	1
3	C_1_	100	1	10

### 2.6. Electrode preparation

The active material, *i.e.*, MOF, was combined with acetylene black, 2-pyrrolidone (NMP), and polyvinylidene fluoride (PVDF) binder in 8 : 1 : 1 relative ratio. This mixture was placed in a septa vial and stirred for a duration of 8 hours until a homogeneous slurry was obtained. A Ni foam substrate with a surface area of 1 cm × 1 cm was subjected to a cleaning process involving sequential washes with HCl, ethanol, and acetone. Subsequently, it was further washed, and probe sonicated for 10 minutes in deionized (DI) water, serving as a current collector. The prepared slurry was then deposited onto the cleaned Ni foam substrate using a micropipette, with a dropwise manner of deposition and dried at 80 °C for 24 hours. The estimated active mass of the loaded material was 6 mg for both three as well as two electrode assemblies. A total of six electrodes were prepared, representing the MOFs, PANI, rGO, C_1_, C_2_ and C_3_ compositions.

### 2.7. Device preparation

The C_2_ composition was employed as the positive electrode, while the negative electrode consisted of activated carbon (AC). Whatman filter paper no. 1 served as the separator and a 1 M KOH solution was utilized as the electrolyte. The electrode configuration employed in this study signifies an asymmetric nature of the device, using a two-electrode assembly. The fabrication process for the AC electrode followed the same procedure as the other electrodes. The calculation of electrochemical parameters during testing involved the utilization of the following equations in Table 2.^33^

**Table 2:** List of formulae used to extract results from data

Equation no.	Equation	Terms
1	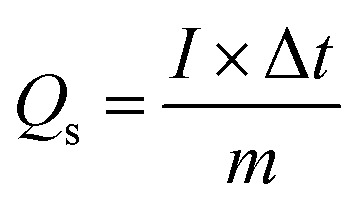	*Q*_s_ is the specific capacity (C g^−1^), *I* is the charging current (A), *m* is the mass of the electrode material (g), and Δ*t* is the discharge time in GCD
2	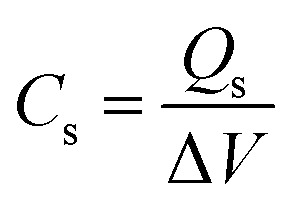	*C*_s_ is the specific capacitance (F g^−1^), *Q*_s_ is the specific capacity (C g^−1^), and Δ*V* is the operating potential window in GCD
3	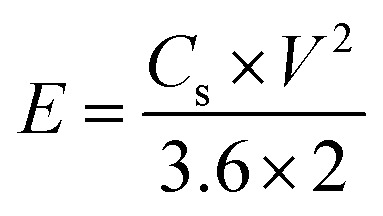	*C*_s_ is the specific capacitance (F g^−1^), *E* is the energy density (W h kg^−1^), and *V* is the operating voltage (V)
4	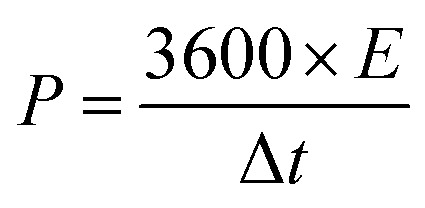	Δ*t* is the discharge time (*t*) and *E* is the energy density (W h kg^−1^)

### 2.8. Characterization

The synthesized materials were subjected to comprehensive characterization using advanced analytical techniques. The crystallographic properties were determined employing Proto's X-ray diffractometer, procured from the United Kingdom, and equipped with *λ* = 1.5418 nm Cu-Kα radiation source. The XRD measurements were conducted across 10° to 60° of the 2*θ* diffraction angle range. The corresponding X-ray diffraction intensity *vs.* 2*θ* data was recorded. The microstructural analysis was carried out utilizing ZEISS's scanning electron microscopy, which was procured from the United Kingdom. The electrochemical evaluation of the synthesized materials was performed to investigate their electrochemical performance. For this, a Gamry Instruments' Galvanostat potentiostat (Reference 3000), procured from the United States, was used. As a result, the electrode's as well as devices' cyclic voltammetry (CV), galvanostatic charge/discharge (GCD) tests, impedance, and stability assessment were conducted. The electrode testing involved a three-electrode assembly, with a platinum (Pt) wire and a Hg/HgO, serving as the reference and counter electrodes, respectively. The device as well as working electrodes were synthesized utilizing Ni-foam, as previously explained in the experimental procedure.

## 3. Results and discussion

### 3.1. Crystallography and microstructure analysis

**Fig. 2:**
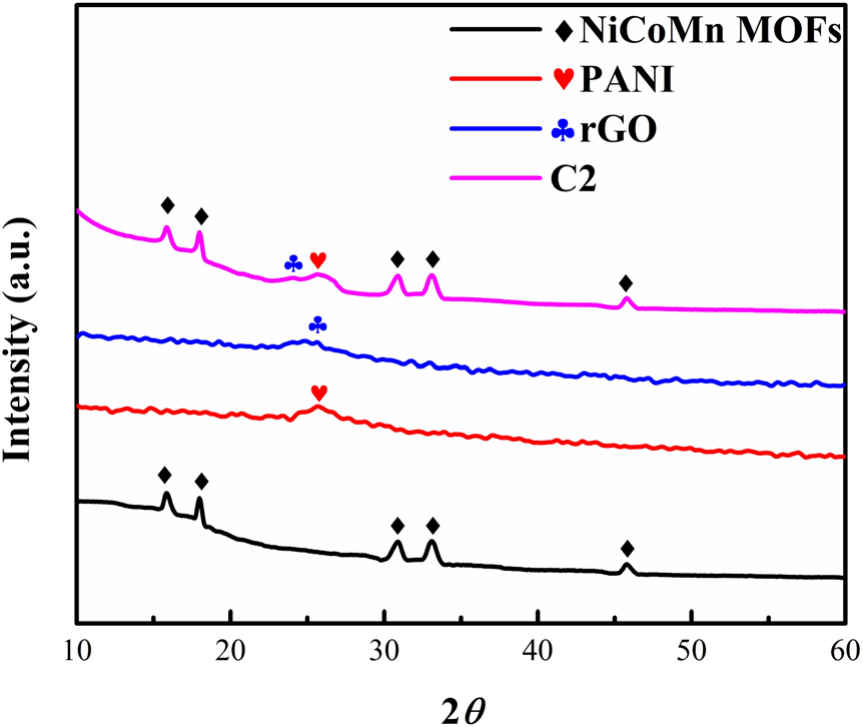
The XRD measurements of Ni–Co–Mn MOFs, PANI, and rGO electrodes over a 10° to 60° range of 2*θ* diffraction angle.

The X-ray diffraction (XRD) analysis revealed distinct peaks of metal–organic framework (MOF) and reduced graphene oxide (rGO) samples, and polyaniline (PANI) sample, as shown in Fig. 2. The MOF exhibited prominent peaks at 15.85°, 18.02°, 30.741°, 33.06°, and 45.3°. The first two peaks at 15.85° and 18.02° have been reported in the literature and are considered characteristic of Ni-based MOFs,^45^ the peaks at 30.71°, and 33.06° are associated with Co-based MOFs indicating the presence of Co in the MOF^46,47^ and the peak at 45.3° corresponds to the presence of Mn in the MOF.^48^ A distinct peak at 25.08° was observed, which corresponds to the characteristic peak associated with the formation of rGO.^49^ The presence of a peak at 25.7° in the PANI sample, closely matching the literature-reported value, serves as a characteristic indication of successful polyaniline synthesis.^50^ The material of interest, *i.e.*, C_2_ nanocomposite, showed the characteristic peaks of Ni–Co–Mn MOFs, PANI, and rGO, indicating its composite nature.

**Fig. 3:**
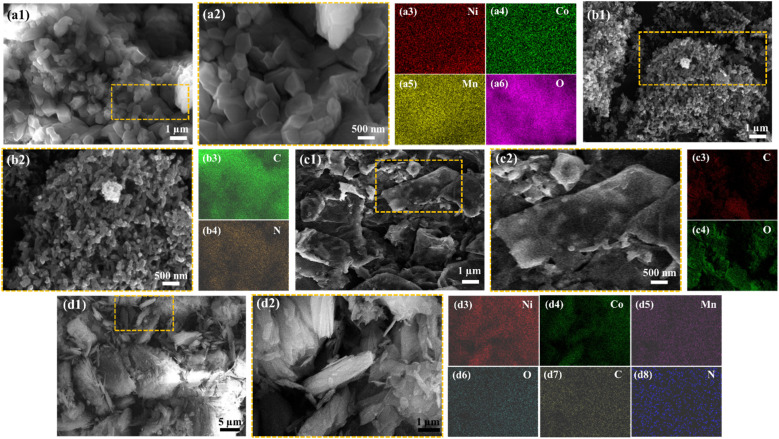
Low magnification SEM micrographs of NiCoMn MOFs (a1), PANI (b1), rGO (c1), and the material of interest, *i.e.*, C_2_ (d1). High magnification SEM micrographs of NiCoMn MOFs (a2), PANI (b2), rGO (c2), and the material of interest, *i.e.*, C_2_ (d2). Elemental area mapping of constituent elements of NiCoMn MOFs (a3–a6), PANI (b3 and b4), rGO (c3 and c4), and the material of interest, *i.e.*, C_2_ (d3–d8).

Fig. 3(a1)–(a6) shows the SEM micrographs as well as EDS elemental area mapping of the Ni–Co–Mn MOFs. It reveals its distinct cubic morphology,^46^ which is consistent with previous findings that associate Co with such structural features in MOFs. The EDS elemental area mapping performed on the MOFs sample confirms the uniform distribution of nickel (Ni), cobalt (Co), and manganese (Mn) elements throughout the material. The even dispersion of these elements signifies a homogeneous distribution within the MOF, supporting its overall compositional integrity. The SEM image of reduced graphene oxide rGO, see Fig. 3(b1) and (b2) displays a distinct fibrous-like structure with a diameter of around 93 nm. This observation aligns with the expected appearance of rGO, which is known for its two-dimensional, planar structure resembling nanosheets. Fig. 3(b3) and (b4) indicate the EDS elemental area mapping of the rGO sample, revealing an even dispersion of carbon and oxygen elements throughout the material, which further supports the understanding that it is predominantly composed of carbon atoms and oxygen functional groups.

Moreover, in Fig. 3(c1) and (c2), a 1D cross-linked fiber-like microstructure of PANI is demonstrated. In addition, the EDS elemental area mapping performed on the PANI confirms the uniform distribution of carbon (C) and nitrogen (Ni) elements throughout the material. The even dispersion of these elements signifies a homogeneous distribution within the MOF, supporting its overall compositional integrity. More importantly, in Fig. 3(d1) and (d2), *i.e.*, C_2_ the material of interest, the encapsulation process of the cubic MOF crystal by the rGO nanosheets and PANI is prominently demonstrated. This formation results in the development of a composite structure, which is expected to contribute to enhanced specific capacitance properties, as will be elucidated in the subsequent sections of this study. Its elemental area mapping, see Fig. 3(d3)–(d8) indicated the even distribution of all the constituent elements of NiCoMn MOFs, rGO nanosheets, and PANI 1D nanostructures.

### 3.2. Electrochemical analysis of the prepared electrodes in a three-electrode assembly

The base nanomaterials, encompassing Ni–Co–Mn MOFs, PANI, and rGO, along with their corresponding composites denoted as C_1_, C_2_, and C_3_, were subjected to a series of electrochemical techniques, namely cyclic voltammetry (CV), galvanostatic charge–discharge (GCD), and electrochemical impedance spectroscopy (EIS). Firstly, cyclic voltammetry (CV) using a three-electrode configuration was used to characterize the redox behaviour and assess the tendency for electric double-layer capacitance (EDLC) and pseudocapacitance of all the aforementioned materials. A range of scan rates, spanning from 2 mV s^−1^ to 50 mV s^−1^ in a potential window from 0–0.7 V. The area under the curve increases with the increase in scan rate, which can be attributed to a higher potential applied in a second, stimulating a higher oxidation current peak. This behaviour is observed in all the tested materials and is characteristic due to reaction kinetics in the solution.

**Fig. 4:**
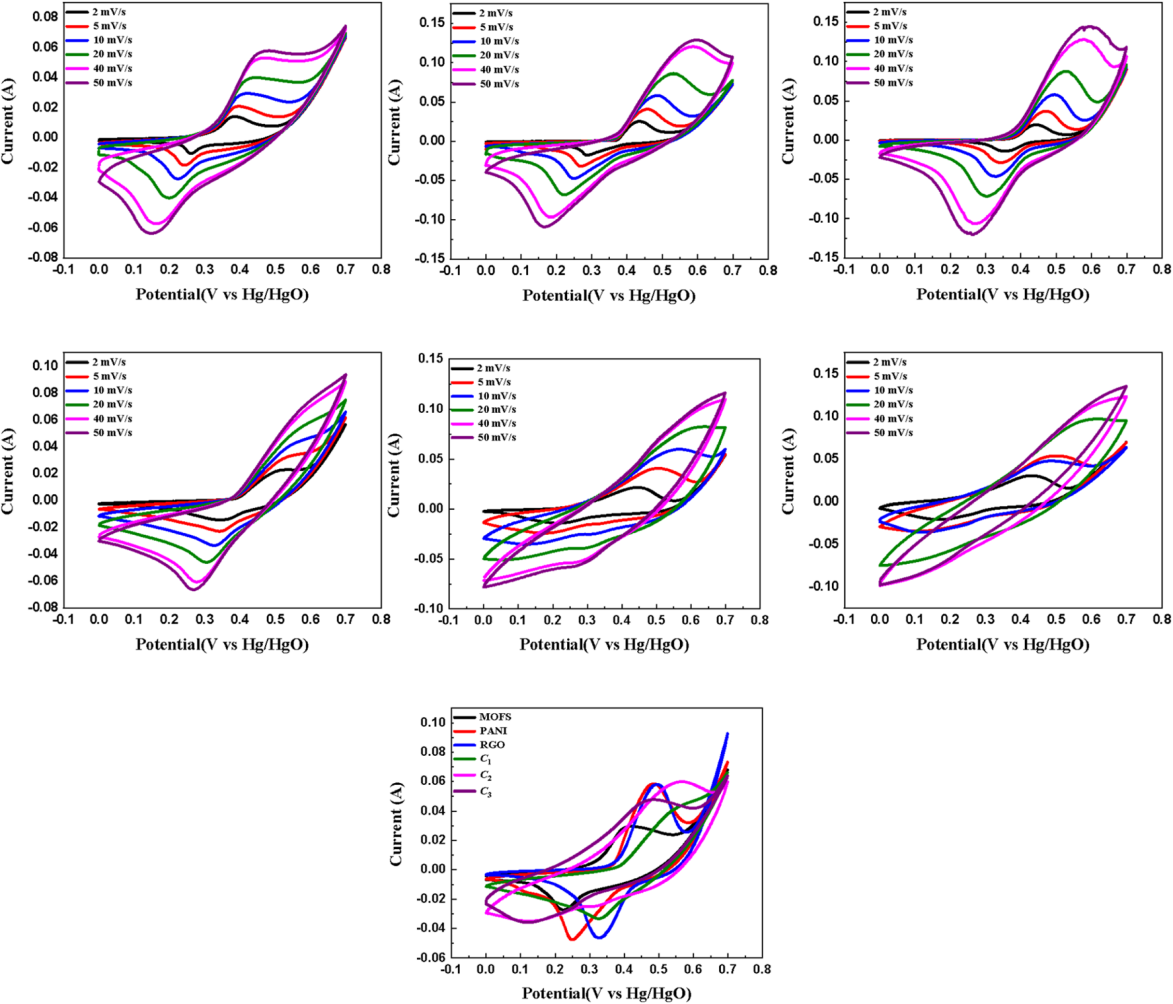
CV characterization of NiCoMn MOFs (a), PANI (b), rGO (c), C_1_ (d), C_2_ (e), C_3_ (f) at scan rates 2–50 mV s^−1^ and comparative curves of all the aforementioned materials at a scan rate of 10 mV s^−1^ (g).

As previously indicated, metal–organic frameworks (MOFs) have demonstrated remarkable performance in supercapacitor applications, primarily owing to their noteworthy attributes of high surface area and exceptional porosity. These characteristics afford MOFs the capacity to exhibit both electric double-layer capacitance (EDLC) behaviour, facilitated by their high surface area and porosity, as well as pseudocapacitance arising from the variable oxidation states of the transition metals embedded within their structures.^51,52^ The observed behaviour, with a relatively low peak current and area under the curve, is clearly depicted in the curves presented in Fig. 4(a). The shape of the CV curve exhibited by the MOFs in the cyclic voltammetry (CV) analysis approximates a rectangular shape, which is indicative of the Electric Double-Layer Capacitance (EDLC) behaviour while the presence of redox peaks indicates the propagation of a faradaic reaction as well. This implies the occurrence of a reaction with the potential for enhancing the faradaic efficiency. The low intensity of the redox peaks signifies the need for improvements in charge storage kinetics within the material.

The observed shapes of the peaks depicted in Fig. 4(b) and (c) indicate the prevalence of pseudocapacitive behaviour during the testing of rGO and PANI. This is evident from the distinctive curves and the appearance of oxidation and reduction peaks at 0.486 V/0.061 A and 0.250 V/−0.048 A for PANI, as well as at 0.498 V/0.061 A and 0.327 V/−0.046 A for rGO. Comparatively, the discrepancy in the values between oxidation and reduction peaks is smaller for rGO and PANI when compared to pristine MOFs, implying that both rGO and PANI exhibit higher faradaic efficiency than MOFs. While reduced graphene oxide (rGO) commonly shows electric double-layer capacitance (EDLC) behaviour based on literature, our study found pseudocapacitive behaviour in rGO. This is likely due to the incomplete removal of oxygen-containing functional groups in graphene oxide (GO) during thermal reduction and the formation of structural defects during thermal decomposition. The remaining functional groups can introduce redox-active sites, contributing to observed pseudocapacitive behaviour. Thermal decomposition can create defects like vacancies and edges, acting as localized regions for additional charge storage.^53^

The cyclic voltammetry (CV) curves exhibited by the composites depicted in Fig. 4(d)–(f) demonstrate a hybrid behaviour characterized by both electric double-layer capacitance (EDLC) and pseudocapacitance. The oxidation and reduction peaks of C_1_ indicate a behaviour leaning towards pseudocapacitive with values 0.573 V/0.048 A and 0.329 V/−0.033 A respectively. Notably, C_2_ shows an oxidation peak discernible at 0.562 V/0.0636 A when employing a scan rate of 10 mV s^−1^, suggesting the presence of hybrid behaviour. The corresponding reduction peak is observed at 0.147 V/−0.0365 A. These findings suggest a favourable faradaic efficiency for C_2_. In contrast, the oxidation peak of C_3_ at 0.480 V/0.047 A is more prominent than the reduction peak at 0.126 V/−0.036 A, indicating relatively lower ease of electron transfer for charge storage in this composite. The integrated area under the CV curve provides insights into the energy storage capacity of the composites. Among the three composites, C_2_ exhibits the largest area under the curve, suggesting its ability to store the highest amount of energy, followed by C_3_ and then C_1_. These observations are further confirmed by the galvanostatic charge–discharge (GCD) analysis, which will be elaborated upon in the subsequent section. Fig. 4(g) presents the CV scans of all the materials at a scan rate of 10 mV s^−1^, indicating a relatively high discharge time for our material of interest *i.e.*, C_2_ nanocomposite.

**Fig. 5:**
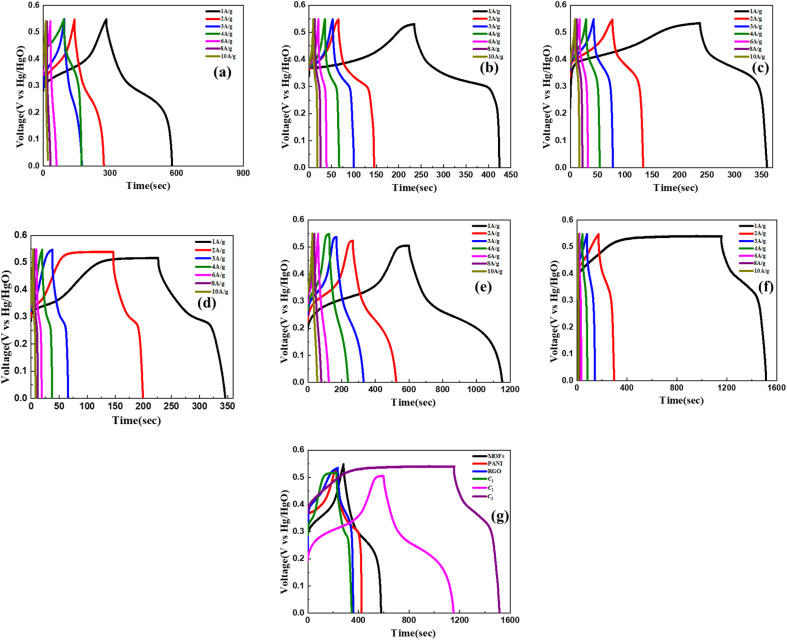
Discharge curves from GCD characterization of NiCoMn MOFs (a), PANI (b), rGO (c), C_1_ (d), C_2_ (e), C_3_ (f) at current densities 1–10 A g^−1^ and comparative curves of all the aforementioned materials at a current density of 1 A g^−1^ (g).

The GCD analysis was conducted under identical experimental conditions as those mentioned previously for the CV analysis. The potential window applied during the GCD analysis ranged from 0 to 0.55 V, while the charging and discharging current density were maintained at equivalent levels. Notably, in all the presented figures, the discharge profiles exhibited a non-linear characteristic, suggesting the presence of a hybrid behaviour. This observation aligns consistently with the results obtained from the CV analysis, agreeing to the coexistence of both EDLC and pseudocapacitive contributions within the investigated materials. From eqn (1) it is evident that at a current density of 1 A g^−1^, the discharge time corresponds to the specific capacity of the system. From Fig. 5(a)–(c) it can be observed that Ni–Co–Mn MOFs exhibit a specific capacitance of 217.2 F g^−1^, whereas rGO and PANI exhibit 224 F g^−1^ and 345.4 F g^−1^, respectively.

The obtained results of the GCD analysis for the composites depicted in Fig. 5(d)–(f) demonstrate that C_2_ exhibits superior performance compared to the other composites. This is evidenced by its specific capacitance of 1104 F g^−1^ at 1 A g^−1^, whereas C_1_ shows a specific capacitance of 538.7 F g^−1^ and C_3_ attains a value of 720 F g^−1^. The decrease in performance of nanocomposite C_1_ in comparison to C_2_ and C_3_ is likely caused by the addition of insignificant amounts of PANI and rGO to MOFs. This effect arises from disturbances in the linker bonds caused by the presence of PANI and rGO, without a significant enhancement in conductivity due to the limited quantity of these materials. Consequently, the surface area of the MOFs is reduced. This phenomenon can be attributed to the interference of rGO with the ligand molecules, resulting in bond perturbations. Additionally, the presence of rGO in small quantities is insufficient to effectively enhance the conductivity of the composite, while also contributing to the blockage of pores within the MOFs.^54^ Upon the introduction of an adequate quantity of rGO and PANI, an intriguing phenomenon arises wherein the rGO sheets enshroud the block-like architecture of MOFs, imparting a box-like configuration. Concurrently, PANI assumes the role of an adhesive binder, facilitating the connection between the two constituents. This particular structural arrangement not only promotes the synergistic integration of charge storage capabilities from both materials but also establishes a conductive network encompassing the exterior surface of the MOFs. As a consequence, an enhancement in performance is achieved. The absence of this structural arrangement in C_1_ can be attributed to an insufficient number of rGO sheets available to envelop the MOFs. Conversely, C_2_ and C_3_ exhibit this characteristic structure, thereby demonstrating the anticipated effects on performance. Fig. 5(g) presents the GCD curves of all materials under investigation, specifically at an applied current density of 1 A g^−1^. This comparison is intended to facilitate a comprehensive assessment and comparative analysis of their electrochemical performance.

**Fig. 6:**
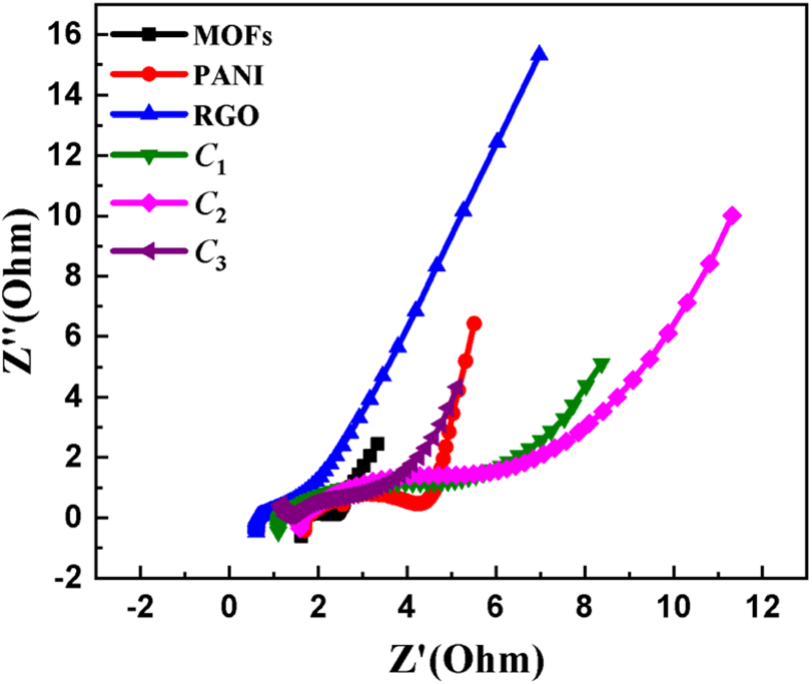
EIS analysis results characterizing as Nyquist plot demonstrating the internal resistances of NiCoMn MOFs, PANI, rGO, C_1_, C_2_, C_3_.

The Nyquist plot derived from the analysis of electrochemical impedance spectroscopy (EIS) for the materials under investigation is presented in Fig. 6. The EIS measurements provide quantitative insights into crucial electrode parameters, notably the electrical resistance. The EIS Nyquist plots, which consist of a low-frequency linear segment and a high-frequency semicircular section, allow for the observation of faradaic processes over a frequency range spanning from 100 mHz to 100 kHz. For the pristine materials (such as MOFs, PANI, and rGO), a negligible charge transfer resistance (*R*_ct_) was observed as evidenced by the absence of a discernible semicircular feature. This observation differs from composites, which exhibit higher *R*_ct_ due to their lower conductivity compared to highly conductive materials like PANI and rGO, see Table 3. The ionic resistance (*R*_s_) of the electrolyte at the point of interaction in the high-frequency region with the real impedance (*Z*′, Ω) axis remains consistently low across the board. Within the lower frequency range, the curved trace denoting Warburg impedance (*W*) indicates a near 45° angle for samples containing varying amounts of rGO (C_1_, C_2_, C_3_), suggesting hybrid behaviour that leans toward electric double-layer capacitors (EDLC) with an increasing amount of rGO. On the other hand, the standard MOFs and PANI exhibit behaviour consistent with pseudocapacitive materials, while rGO displays a hybrid behaviour rather than EDLC due to the presence of unreduced oxygen, as previously explained.

**Table 3:** Different resistance values of five electrodes employing EIS-fitted solution

Electrode materials	*R*_s_ (ohms)	*R*_ct_ (ohms)	*W* (ohms)
MOFs	1.631	0.7765	0.04308
PANI	1.637	3.294	0.03675
rGO	0.6166	2.026	0.1748
C_1_	1.096	5.548	0.05513
C_2_	1.543	7.007	0.04501
C_3_	1.393	2.274	0.5644

**Fig. 7:**
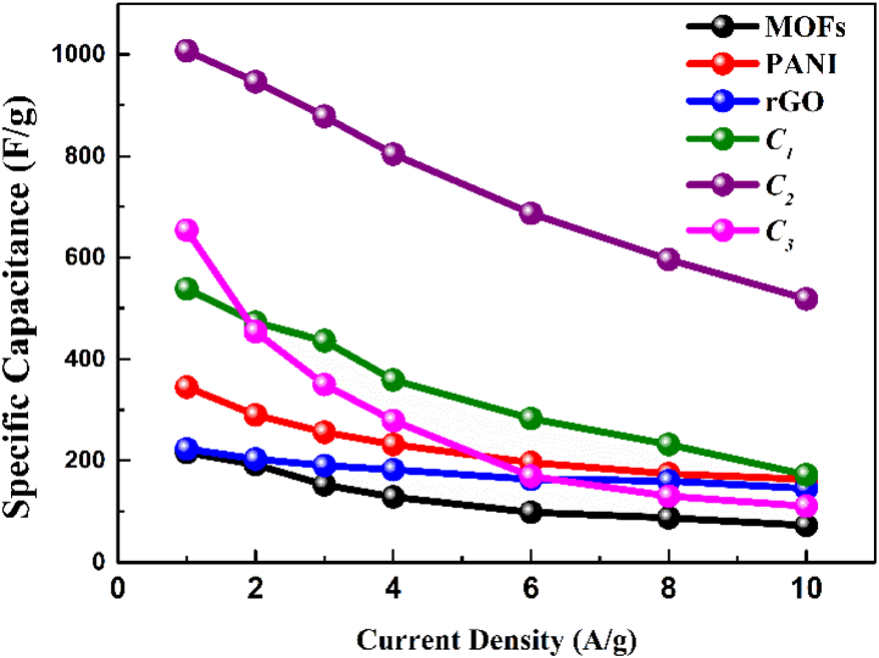
Comparison of specific capacitances at several current densities.

As shown in Fig. 7, C_2_ demonstrates the specific capacitances *vs.* the current density of three electrodes. It showcased a peak capacitance of 1007 F g^−1^ (at 1 A g^−1^), derived from eqn (2). The observed decrement in specific capacitance values was attributed to the constrained time window available for electrolytic ions to engage in encounters and diffusion throughout the electrode structure. Remarkably, the electrodes, most notably C_2_, maintained their specific capacitance even under elevated current densities (518 F g^−1^ at 10 A g^−1^), and also showed much better performance across the board as illustrated, thus attesting to its exceptional capacitance retention.

Table 4 serves as a means to assess and compare the merit of this work in comparison to the already reported literature. Ni/Co-MOF-rGO, fabricated *via* the process of stir-heating, revealed a specific capacitance of 860 F g^−1^ at 1 A g^−1^ in a 6 M KOH environment, as documented in ref. 55. Similarly, NiCo-MOF/AB-5, prepared through ultrasonication, yielded a specific capacitance of 916.1 F g^−1^ at 1 A g^−1^ in 2 M KOH.^56^ The dandelion-like Ni/Co-MOF, synthesized hydrothermally, yielded a specific capacitance of 758 F g^−1^ at 1 A g^−1^ in 2 M KOH, in accordance with ref. 57 The Co/Mn–MOF, synthesized using a hydrothermal technique, demonstrated a specific capacitance of 1176.59 F g^−1^ at 3 mA cm^−2^ in 1 M KOH, as described in ref. 58 Lastly, the present study, encompassing C_2_ (MOFs : rGO : PANI mass ratio of 100 : 1 : 1), was produced through a hydrothermal method, resulting in a specific capacitance of 1007 F g^−1^ at 1 A g^−1^ in 1 M KOH, signifying a recent and notable advancement in this research domain.

**Table 4:** The comparison of present work with literature

Materials	Method of synthesis	Specific capacitance	Electrolyte	Ref.
Ni/Co-MOF-rGO	Stir-heating	860 F g^−1^ at 1 A g^−1^	6 M KOH	55
NiCo-MOF/AB-5	Ultrasonication	916.1 F g^−1^ at 1 A g^−1^	2 M KOH	56
Dandelion like Ni/Co-MOF	Hydrothermal	758 F g^−1^ at 1 A g^−1^	2 M KOH	57
Co/Mn–MOF	Hydrothermal	1176.59 F g^−1^ at 3 mA cm^−2^	1 M KOH	58
Ni–Co–Mn MOFs	Hydrothermal	217.2 F g^−1^ at 1 A g^−1^	1 M KOH	This work
C_2_ (MOFs : rGO : PANI mass ratio of 100 : 1 : 1)	Hydrothermal	1007 F g^−1^ at 1 A g^−1^	1 M KOH	This work

### 3.3. Asymmetric two-electrode supercapacitor device's electrochemical analysis

**Fig. 8:**
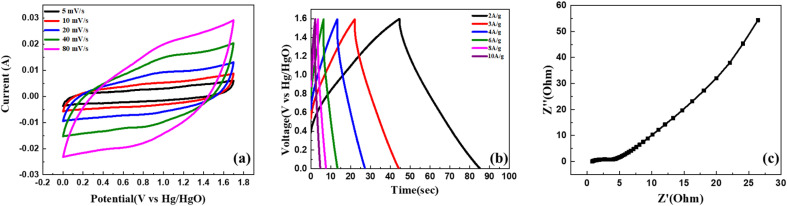
CV (a), GCD (b), and EIS (c) analysis results of the asymmetric supercapacitor device.

An asymmetric supercapacitor device was developed by utilizing activated carbon (AC) as the negative electrode and C_2_, the most efficient composite sample, as the positive electrode. The electrodes were immersed in a 1 M KOH solution, serving as the electrolyte. We conducted cyclic voltammetry (CV) analysis in a potential window ranging from 0 V to 1.7 V while varying the scan rates from 1 mV s^−1^ to 100 mV s^−1^. The obtained CV curves, as shown in Fig. 8(a), exhibited a quasi-rectangular shape at different scan rates. This distinctive feature indicates the presence of both electric double-layer capacitance (EDLC) and pseudo-capacitance behaviour in the system, contributing to a hybrid performance of asymmetric supercapacitor (ASC) device. Notably, the shape of CV curves remained undistorted even with the increment in scanning rate, providing evidence of strong reversibility and excellent overall performance of the ASC.

The GCD (galvanostatic charge–discharge) analysis was performed within a potential window of 1.6 V, and the scan rates were varied from 1 A g^−1^ to 10 A g^−1^, as illustrated in Fig. 8(b). The data point obtained at 1 A g^−1^ was utilized to derive the energy and power density of the device using eqn (3) and (4). The calculated results yielded an energy density of 25.11 W h kg^−1^ and a power density of 860 W kg^−1^. The electrochemical impedance spectroscopy (EIS) analysis revealed minimal resistance in the device. The ASC device has an *R*_s_ and *R*_ct_ value of 0.6735 Ω and 57.98 Ω, respectively, indicating a decent contact at the electrode/electrolyte interface and desirable capacitive behaviour as shown in the Nyquist plot illustrated in Fig. 8(c). These observations collectively indicate the excellent conductivity and efficient charge transport properties of the device, contributing to its favourable electrochemical performance.

**Fig. 9:**
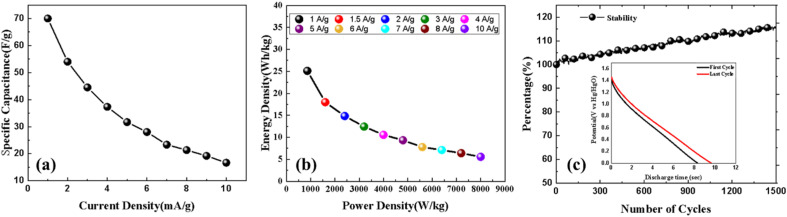
The device's current density-varied specific capacitance (F g^−1^) (a), its Ragone plot (b), and its cyclic stability at a current density of 6 A g^−1^ for 1500 cycles (c).

Fig. 9(a) shows the trend in specific capacitance as the current density of the system varies from 1 A g^−1^ to 10 A g^−1^. The corresponding Ragone plot is shown in Fig. 9(b). For practical applications of this material in daily use, it becomes imperative to assess its cyclic stability. In this regard, we subjected the device to 1500 charge and discharge cycles at a rate of 6 A g^−1^. The results, presented in Fig. 9(c), revealed a remarkable behaviour, wherein the capacitance not only retained its initial value but even exhibited an increase, reaching up to 115% of its initial value. This substantial enhancement in capacitance demonstrates the excellent cyclic stability of the device. Additionally, the improved cyclic stability is ascribed to the device's enhanced wettability with the electrolyte ions during the charge discharge process, resulting in an increase in the number of active charge storage sites.^59^ This phenomenon facilitates efficient charge storage and delivery throughout the repeated cycles, contributing to the sustained and enhanced capacitance observed in the device. Such outstanding cyclic stability holds significant promise for the reliable and long-lasting utilization of this material in various practical applications.

The examination of diffusive and capacitive behaviour involved the extraction of *b* values from the CV curves, employing the equation of power law as expressed by eqn (5):

5 *i* = *av*^*b*^

In this equation, the symbol *b* represents the gradient value, while the parameters *a* and *b* serve as adjustable factors. In this context, *i* relates to the electric current, and *v* denotes the rate of scanning. By applying a logarithmic transformation to both sides of the equation, we derive eqn (6):

6 log(*i*) = log(*a*) + *b* log(*v*)

**Fig. 10:**
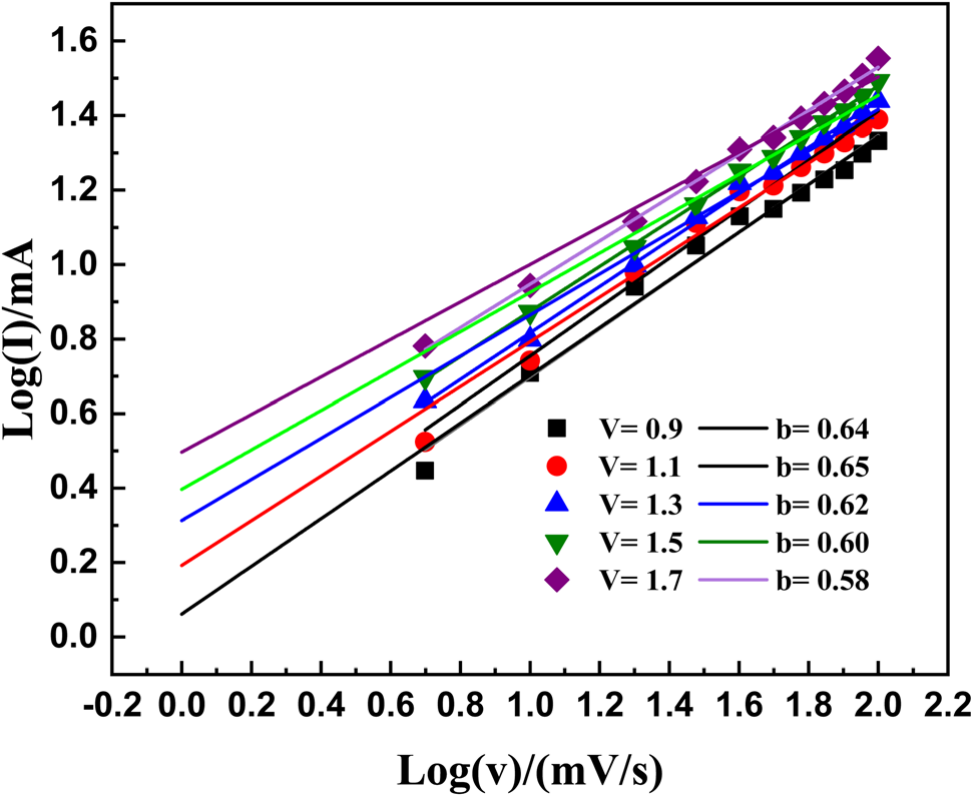
Illustrates the linear fitting of the logarithm of current (log(*i*)) plotted against the logarithm of voltage (log(*v*)) at various potentials for the computation of the *b*-values.

The parameter *b* covers different intervals: (i) a value of 1 indicates a supercapacitor, (ii) a value of 0.5 indicates a material of battery-grade calibre, and (iii) a value ranging from 1 to 0.5 indicates a mixed characteristic within the device.^25,60^ The complete assessment of *b* coefficients is visually represented in Fig. 10. This methodical examination unveils *b* coefficients spread across the range of 0.5 to 1; see Fig. 10. It highlights the simultaneous existence of both non-faradaic and faradaic responses. The identified *b* coefficients support our claim of a combined supercapacitor configuration.

## 4. Conclusion

The study introduced a new nanocomposite containing Ni–Co–Mn metal–organic frameworks (MOFs), polyaniline (PANI), and reduced graphene oxide (rGO) for supercapacitor charge storage improvement. Through comprehensive material analysis and ratio variations, composition C_2_ (MOFs : rGO : PANI mass ratio of 100 : 1 : 1) emerged as the most promising, displaying outstanding electrochemical performance. The C2 electrode, utilized in a 1 M KOH electrolyte, achieved a specific capacitance of 70.625 F g^−1^ at 1 A g^−1^, 25.11 W h kg^−1^ energy density, and 860 W kg^−1^ power density. Impressively, after 1500 cycles, the asymmetric supercapacitor displayed 115% capacity retention, thanks to improved electrode surface wettability and pore re-opening. Additionally, the observed *b* values support the claim of a hybrid supercapacitor, further emphasizing the significant potential of the developed nanocomposite composition in enhancing charge storage capabilities for supercapacitor applications. The study underscores the potential of this nanocomposite for high-performance, durable supercapacitor applications, advancing energy storage capabilities.

## Data availability

The data that has been used is confidential.

## Author contributions

Mohsin Ali Marwat: conceptualization, validation, formal analysis, resources, writing – original draft, supervision, project administration, funding acquisition, and submission to journal. Shaheer Ishfaq: synthesis, writing – original draft, electrochemical analysis and revisions. Kanwar Muhammad Adam: synthesis, data curation, writing – original draft, and writing – review & editing. Bilal Tahir and Muhammad Hamza Shaikh: electrochemical analysis and revision file preparation. Muhammad Fawad Khan: writing – original draft. Muhammad Ramzan Abdul Karim: validation and resources. Zia Ud Din, Syed Abdullah and Esha Ghazanfar: writing – original draft and writing – review & editing.

## Conflicts of interest

The authors declare that they have no known competing financial interests or personal relationships that could have appeared to influence the work reported in this paper.

## Supplementary Material
